# Neurology: A Better Model for PD

**Published:** 2004-12

**Authors:** Hakon Heimer

The ubiquitin–proteasome system mediates protein recycling by tagging abnormal or unwanted proteins within cells with the small protein ubiquitin. Enzymes called proteasomes then dismantle the tagged proteins. Malfunctioning of this system is emerging as an important component of neurodegenerative diseases, such as Parkinson disease (PD), that feature the buildup of defective proteins and the gradual death of brain cells. This new PD research focus was validated by a recent study in which researcher Kevin St. P. McNaught and colleagues at the Mount Sinai School of Medicine induced a disorder closely resembling PD by exposing rats to proteasome inhibitors.

Researchers have long been able to create PD models in laboratory animals by using toxicants that kill dopamine nerve cells in an area of the brain called the substantia nigra. The substantia nigra is an important node in the brain circuitry that controls movement, and neurons in this area are hardest hit by PD. But in neurotoxicant models, the animals do not develop the full range of clinical and pathological features of the human disease, especially those that result from nerve cell death in other brain regions.

In their quest for a more representative model of PD, McNaught and colleagues took note of recent evidence that malfunction of the ubiquitin–proteasome system is a central factor in both the rare hereditary and common sporadic forms of PD. Over a period of two weeks, they injected rats with both man-made and naturally occurring proteasome inhibitors. Within two weeks of exposure, the rats began to show parkinsonian symptoms, including slowness of movement, rigidity, and tremor. “These symptoms gradually worsened over a period of weeks to months, and could be reversed with drugs that are used to treat PD patients,” says McNaught.

PET imaging and autopsy studies of the animals’ brains showed changes very similar to those seen in PD, including the abnormal accumulation of protein in the substantia nigra. In response to the proteasome inhibitors, nerve cells all over the brain boosted their proteasomal activity, but the substantia nigra and other PD-affected areas were unable to sustain this compensatory response, and ultimately showed reduced proteasomal activity as occurs in PD.

In the report of their findings, which was published in the July 2004 issue of the *Annals of Neurology*, the authors also raise the possibility that proteasome inhibitors in the environment, whether from bacteria, fungi, plant-based foods, or man-made sources, might play a role in the development of some cases of PD. Importantly, recent studies have shown that the widely used fungicide maneb is a potent proteasome inhibitor and can kill dopamine cells in culture. We must therefore determine the extent to which proteasome inhibitors are present in the environment and how humans might be exposed to these agents, says McNaught.

Jean Harry, principal investigator of the NIEHS Neurotoxicology Group, believes such a link is tenuous at this stage. She says, “What the study really offers the field is an exciting new model to address questions about the disease process and the potential impact of environmental factors.”

